# Insights into treatment and outcome of fracture-related infection: a systematic literature review

**DOI:** 10.1007/s00402-018-3048-0

**Published:** 2018-10-20

**Authors:** H. Bezstarosti, E. M. M. Van Lieshout, L. W. Voskamp, K. Kortram, W. Obremskey, M. A. McNally, W J. Metsemakers, M. H. J. Verhofstad

**Affiliations:** 1 Trauma Research Unit Department of Surgery, Erasmus MC, University Medical Center Rotterdam, P.O. Box 2040, 3000 CA Rotterdam, The Netherlands; 2 Vanderbilt University, Nashville, USA; 3 Nuffield Orthopaedic Centre, Oxford University Hospitals, Oxford, UK; 4 Department of Trauma Surgery, University Hospitals Leuven, Leuven, Belgium

**Keywords:** Fracture-related infection, Outcome measurements, Diagnosis, Treatment, Definition, Classification

## Abstract

**Introduction:**

Standardized guidelines for treatment of fracture-related infection (FRI) are lacking. Worldwide many treatment protocols are used with variable success rates. Awareness on the need of standardized, evidence-based guidelines has increased in recent years. This systematic literature review gives an overview of available diagnostic criteria, classifications, treatment protocols, and related outcome measurements for surgically treated FRI patients.

**Methods:**

A comprehensive search was performed in all scientific literature since 1990. Studies in English that described surgical patient series for treatment of FRI were included. Data were collected on diagnostic criteria for FRI, classifications used, surgical treatments, follow-up protocols, and overall outcome. A systematic review was performed according to the PRISMA statement. Proportions and weighted means were calculated.

**Results:**

The search yielded 2051 studies. Ninety-three studies were suitable for inclusion, describing 3701 patients (3711 fractures) with complex FRI. The population consisted predominantly of male patients (77%), with the tibia being the most commonly affected bone (64%), and a mean of three previous operations per patient. Forty-three (46%) studies described FRI at one specific location. Only one study (1%) used a standardized definition for infection. A total of nine different classifications were used to guide treatment protocols, of which Cierny and Mader was used most often (36%). Eighteen (19%) studies used a one-stage, 50 (54%) a two-stage, and seven (8%) a three-stage surgical treatment protocol. Ten studies (11%) used mixed protocols. Antibiotic protocols varied widely between studies. A multidisciplinary approach was mentioned in only 12 (13%) studies.

**Conclusions:**

This extensive literature review shows a lack of standardized guidelines with respect to diagnosis and treatment of FRI, which mimics the situation for prosthetic joint infection identified many years ago. Internationally accepted guidelines are urgently required to improve the quality of care for patients suffering from this significant complication.

## Introduction

Fracture-related infection (FRI) is a serious complication related to musculoskeletal trauma. It can have a devastating impact on a patient’s quality of life and has huge socioeconomic consequences [1]. Patients are often not only unable to participate in social activity due to their limited mobility and function. They also encounter higher direct and indirect health care costs. The results from a recent cost analysis showed that the hospital-related health care costs of infected cases are approximately 6.5-times higher than for non-infected cases, which is much higher than data that were previously published [2–4]. These results stress the importance of standardized prevention and treatment guidelines, with respect to this serious complication.

Since the beginning of the twentieth century the cornerstones of treatment have been extensive and multiple debridements with dead space management and soft tissue coverage [5]. Antibiotic therapy was added as technology progressed. Now almost a 100 years later, a wide variety of strategies is used (e.g., 1 stage, 2 stage, Masquelet, RIA, Ilizarov methods, different types of local antibiotics) without clear scientific background, resulting in a wide spread of clinical results. The rate of FRI remains at 20–30%, with a reported overall treatment failure of 4–11% [6–8].

The aim of this review was to give an overview of the diagnostic criteria, classifications, surgical and follow-up protocols, and success rates of all FRI treatment series published over the last decades.

## Methods

This study was written according to the Preferred Reporting Items for Systematic Reviews and Meta-Analyses (PRISMA) statement [9].

### Literature search strategy

A comprehensive search was performed with the help of a biomedical information specialist in October 2016 and updated on July 1 2017, using Medline, Embase, Web of Science, Cochrane, and Google Scholar. The search strings are recorded in Appendix 1. All studies that described surgical patient series for treatment of FRI were included. Series needed to be greater than five patients and reported in English. Publications before 1990, studies that did not describe FRI patient treatment, and publications reporting non-original data (e.g., reviews or meta-analyses) were excluded. Inclusion consisted of two phases. During the first phase title and abstract were screened for relevance, and full text articles were obtained. When a full text was not available, the corresponding author was contacted once by email. Full text articles were reviewed in the second phase. All references were reviewed by HB and LWV and included after matching the inclusion criteria. Consensus was reached on all references.

### Data extraction

After inclusion, data from each study was independently extracted by two authors (HB and LWV). Disagreements were discussed until agreement was reached. Data were collected in five areas. Part one provided general information from all studies (sample size, age, FRI, and location of FRI). Part two offered information on diagnostic criteria for FRI (given definition of FRI, used classification of FRI, and parameters describing the outcome parameters of FRI).

Part Three focused on data from surgical protocols (number of stages in surgical protocol, type of fixation used, and use of a multidisciplinary approach). Part four described the treatment concept (bone defect size, exact treatment protocol, use of bone, skin or muscle graft, and use of local antibiotics). Part five included the follow-up protocol (bony consolidation without infection after the primary surgical study protocol (primary healing), bony consolidation or amputation without infection at the end of study period (total healing), recurrence of FRI, amputation of the affected limb, number of complications, revision surgery, time to bony union and Hospital Length of Stay (HLOS) and patient reported outcomes).

### Analysis

Results were pooled for the total population presented in the included studies. Binomial data were pooled using Medcalc (MedCalc Statistical Software version 17.9.7) (MedCalc Software bvba, Ostend, Belgium; http://www.medcalc.org; 2017). Heterogeneity was quantified with Cochran’s *Q* test and *I*^2^ statistic, a fixed effects model was used when the *I*^2^ was < 40%. A random effects model was used for the pooled analysis when the heterogeneity test was ≥ 40%. Pooled estimates are reported with their 95% confidence intervals (CI).

Since for continuous data, most studies only provided a mean but not the standard deviation, a full meta-analysis was not feasible. Instead, continuous data were pooled by calculating the weighted mean using Microsoft Excel. Sample size of the individual studies was used as weighting factor. The pooled mean is reported with the range.

## Results

**Fig. 1:**
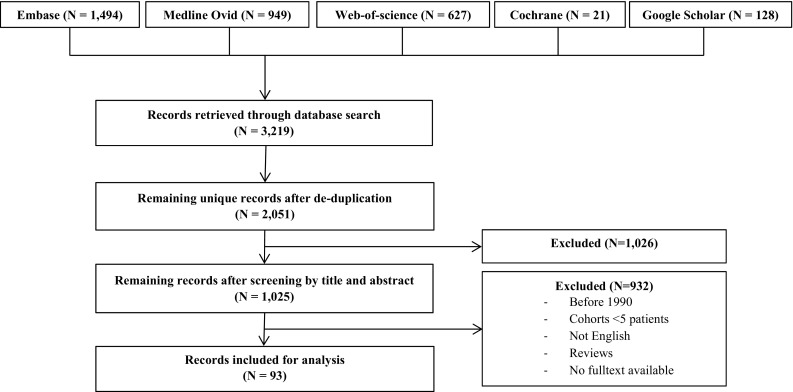
Flowchart of the study

The search identified a total of 2051 unique studies. Figure 1 shows the inclusion flowchart. After selection, 93 studies [8, 10–101] remained for inclusion, describing 3701 patients with 3711 FRI’s. The population consisted predominantly of male patients (*n* = 2656; 77%), with a mean age of 42 (range 6–95) years, a mean infection duration of 28 (range 0–154) months and a mean follow-up of 42 (range 6–101) months. Patients had a mean of 3 (range 0–31) operations before study inclusion. Of all studies, 43 described FRI at one specific location. Table 1 shows the reported locations of FRI, with the tibia being the most commonly affected site (*n* = 2533; 64%), followed by the femur (*n* = 599; 16%).

**Table 1:** Overview of locations of FRI

Location of FRI	All studies, *N* = 3711
Tibia	2533 (68.3%)
Femur	599 (16.1%)
Ankle	124 (3.3%)
Calcaneus	77 (2.1%)
Foot	63 (1.7%)
Humerus	59 (1.6%)
Knee	49 (1.3%)
Fibula	30 (0.8%)
Radius	24 (0.6%)
Forearm	24 (0.6%)
Ulna	18 (0.5%)
Ilium	13 (0.4%)
Elbow	11 (0.3%)
Clavicle	4 (0.1%)
Spine	1 (0.0%)
Skull	1 (0.0%)
Not specified	197 (5.3%)
Lower extremity not defined	41 (1.1%)
Upper extremity not defined	21 (0.6%)

### Diagnosis and classification

An overview of all criteria used to diagnose and/or define FRI is given in Table 2. Diagnostic criteria to define FRI were found in 85 (91%) studies. Clinical (*n* = 83; 89%) and radiological signs (*n* = 73; 78%) were mostly included in the diagnosis of FRI.

**Table 2:** Overview of parameters used to diagnose or define FRI

Parameters associated with FRI	Number of studies, *N* = 93
Clinical signs	
Purulent drainage (or discharge)	34 (37%)
Dolor (pain)	14 (15%)
Tumor (swelling)	9 (10%)
Calor (warmth)	8 (9%)
Rubor (redness)	7 (8%)
Wound dehiscence/breakdown	7 (8%)
Fever	5 (5%)
Unspecified	46 (49%)
Laboratory testing	
C-reactive protein (CRP)	17 (18%)
Unspecified	9 (10%)
Radiological signs	
Signs of osteomyelitis	62 (67%)
Evidence of non-union	34 (37%)
Bacteriological/histological findings	
Cultures taken	56 (60%)
Unspecified histology	17 (18%)
Unspecified bacteriology	15 (16%)
Other	
Time of infection	27 (29%)
No parameters for diagnosis given	8 (9%)

An overview of all described classifications is given in Table 3. A total number of ten different classifications to define infection were found in 47 (51%) studies. The classification of Cierny-Mader was most widely used (*N* = 34; 37%). The duration of the infection was part of infection classifications in 27 (29%) studies. An overview of all time periods recorded in defining FRI can be found in Table 4. Chronic osteomyelitis was described as an infection-period longer than 6 months in four studies (4%), a period of more than 12 weeks in three studies (3%), a period of more than 6 weeks in three studies (3%), and a period longer than 2 months in two studies (2%). Infected non-union was also classified according to time in 12 studies, of which nine (10%) used 6 months as a cutoff.

**Table 3:** Classifications used to define FRI

Classification System	Number of studies, *N* = 47
Cierny-Mader classification	34 (72%)
May’s classification	4 (9%)
Jain and Sinha’s modified May classification	2 (4%)
Calori’s classification for non-union	1 (2%)
CDC guidelines for wound infection	1 (2%)
Osteomyelitis diagnosis score	1 (2%)
UTMB staging system for adult osteomyelitis	1 (2%)
Weiland classification	1 (2%)
Yang’s classification for bone defects	1 (2%)
Ziran’s algorithm for acute infection after ORIF	1 (2%)

**Table 4:** Periods defined in classification of FRI

Time frame	Number of studies *N* = 93
Chronic infection	12 (13%)
> 6 weeks	3 (3%)
> 12 weeks	3 (3%)
> 4 months	2 (2%)
> 6 months	4 (4%)
Infected non-union	12 (13%)
> 3 months	1 (1%)
> 4 months	1 (1%)
> 6 months	9 (10%)
> 12 months	1 (1%)
Multiple divisions	3 (3%)
Acute < 2 weeks, Subacute 2–6 weeks, Chronic > 6 weeks	1 (1%)
Acute < 30 days, Subacute 1–6 months, Chronic > 6 months	2 (2%)
No period of time mentioned	66 (71%)

Finally, three studies (3%) subdivided infection into three time periods (acute, subacute and chronic). Of all studies, 66 (71%), did not mention time in the classification of FRI.

### Surgical protocols

An overview of surgical protocols is given in Table 5. Surgical treatment protocols differed widely across all studies. Overall, 41 (44%) studies described a FRI located in a single anatomic location, compared with 52 (56%) studies, where multiple locations were described.

**Table 5:** Surgical Protocol and Type of Fixation

Number of stages	Number of studies, *N* = 93
One-stage	18 (19%)
Two-stage	50 (54%)
Three-stage	7 (8%)
Combination of the above	10 (11%)
Unknown	16 (17%)

Type of fixation	Number of studies, *N* = 93
Internal	12 (13%)
External	27 (29%)
Bone transport	14 (15%)
Mixed protocols	34 (37%)
Unknown	14 (15%)

A two-stage surgical procedure was used in 50 (54%) studies, followed by a one-stage procedure in 18 (19%), and a three-stage protocol in seven (8%) studies.

Most of the included studies (34; 37%) described a mixture of fracture/bone fixation techniques. Single techniques described were external fixation without bone transport in 27 (29%), followed by external fixation with bone transport in 14 (15%), and internal fixation in twelve (13%). Treatment of bone defects larger than 1 cm were described in 41 (44%) studies.

A multidisciplinary approach in the treatment of FRI was mentioned in 12 (13%) studies, mostly mentioning a team consisting of a trauma or orthopedic surgeon in combination with a plastic surgeon and/or infectious disease (ID) specialist. The different specialties collaborating in such a multidisciplinary team are summarized in Table 6.

**Table 6:** Multidisciplinary approach in treatment of FRI

Specialists involved	Number of studies, *N* = 12
Trauma/orthopedic surgeon	10 (83%)
Infectious disease specialist	5 (42%)
Plastic surgeon	5 (42%)
Pharmacist	1 (8%)
Radiologist	1 (8%)
Team not defined	3 (25%)

### Treatment specifications

An overview of bone grafts used in the treatment of FRI is given in Table 7.

In 62 (67%) studies a total of 1418 bone grafts were described to treat FRI, of which 555 (39%) were cancellous and 381 (27%) were free vascularized bone grafts.

**Table 7:** Bone grafts used in treatment of FRI

Graft type used (62 studies)	*N* = 1418 (% of total)
Cancellous bone	555 (39)
Iliac crest	445 (31)
RIA (with BMP-7)	76 (5)
Tibia condyle	25 (2)
Femur condyle	9 (1)
Vascularized bone	381 (27)
Vascularized fibula	322 (23)
Latissimus dorsi with rib	41 (3)
Vascularized ilium	18 (1)
Other	27 (2)
Graft not defined	455 (32)

Table 8 summarizes all means by which soft tissue reconstruction was achieved. In 41 (44%) studies a total of 1171 methods to reconstruct soft tissue were described, of which 460 (39%) free flaps, 247 (21%) skin grafts, and 125 (11%) rotational flaps. Local antibiotic therapy was used in 51 (55%) studies. Systemic or oral antibiotic treatment regiments differed widely across studies and will be discussed elsewhere.

**Table 8:** Muscle and skin flaps used in treatment of FRI

Total (41 studies)	*N* = 1171 (% of total)
Free flap	460 (39)
Latissimus dorsi	206 (18)
Not defined	82 (7)
Gracilis	64 (5)
Rectus abdominis	47 (4)
Scapular skin flap	23 (2)
Serratus anterior	23 (2)
Lateral thigh	15 (1)
Rotational flap	125 (11)
Gastrocnemius	52 (4)
Saphenous	50 (4)
Soleus	23 (2)
Skin	247 (21)
Split skin graft	232 (20)
Full thickness skin graft	15 (1)
Other	76 (1)
Skin/muscle not defined	200 (17)

### Follow-up protocols

Details on all parameters used in follow-up of FRI can be found in Table 9.

Routine follow-up episodes were defined in 26 (28%) studies, describing clearly defined appointment protocols in 14 (15%) of them. Follow-up parameters used to identify disease recurrence were based on radiology (*N* = 49; 53%), serology (*N* = 32; 34%), and clinical signs (*N* = 31; 33%).

**Table 9:** Parameters used during follow-up of FRI

Parameter	Number of studies, *N* = 93
Predefined routine follow-up episodes	26 (28%)
Clinical signs of infection	31 (33%)
Routine radiological check up	49 (53%)
Blood biomarkers	32 (34%)
C-reactive protein	12 (13%)
Erythrocyte sedimentation rate	10 (11%)
Leucocyte count	4 (4%)
Complete blood count	2 (2%)
Undefined	4 (4%)
Bone scoring systems	5 (5%)
ASAMI	4 (4%)
Bahr score	1 (1%)
Functional outcome scoring systems	19 (20%)
Self-defined functionality scores	5 (5%)
Paley’s functionality score	3 (3%)
American orthopaedic foot and ankle society score	1 (1%)
EuroQol 5D	1 (1%)
Johner-Wruhs evaluation criteria	1 (1%)
Lower extremity functionality scale	1 (1%)
Ma’s knee score	1 (1%)
Mazur’s ankle evaluation grading system	1 (1%)
Merchant and Dietz score	1 (1%)
SF-12	1 (1%)
SF-36	1 (1%)
Shahcheraghi and Bayatpoor evaluation score	1 (1%)
Stewart and Hundley criteria	1 (1%)

Two different scoring systems to describe bone quality were found in five (5%) studies. In 19 (20%) studies a functional outcome scoring system was used, describing 13 different functional outcome scores.

### Surgical outcome

A summary of surgical outcome is detailed in Table 10. Of all 3711 reported complex FRI cases, bone healing and eradication of infection, without recurrence, was reported in 85% (95% CI 82–88) after the primary surgical study protocol. This percentage increased to 93% (95% CI 91–94) if repeated treatment protocols, including amputations, were taken into account. In 3% (95% CI 3–5) of all cases, amputation was deemed necessary to treat FRI. Recurrence of infection was seen in 9% (95% CI 7–11).

**Table 10:** Outcomes of FRI treatment

Proportional variable	Studies (*N*)	FRI (*N*)	Chi^2^ (*p* value)	*I*^2^ value (95% CI)	Pooled proportion (95% CI)
Primary healing	93	3711	^*^	81% (77–84)	85% (82–88)
Total healing	92	3695	^*^	77% (72–81)	93% (91–94)
Infection recurrence	89	3598	^*^	73% (66–78)	9% (7–11)
Amputation rate	83	3226	^*^	50% (35–61)	3% (3–5)

Continuous variable	Studies (*N*)	Patients (*N*)	Weighted mean^b^
Union time (months)	47	1809	6.63
Surgical revisions^a^	58	2110	0.31
Complications^a^	84	3436	0.38
HLOS (months)	14	415	1.39

*p < 0.0001

^a^Per patient

^b^Since most of the individual studies did not report a standard deviation (SD) or standard error, no pooled SD could be provided

A total of 0.39 complications per patient were reported, for which 0.31 surgical revisions per patient were needed. Furthermore, bone healing was achieved in a mean of 7 months (2–15), and patients stayed in the hospital for a mean of 1.39 months (0–3).

## Discussion

To our knowledge this is the first extensive review showing a complete overview of treatment and outcome of FRI from 1990 until 2017. A literature search has been performed, including 93 articles describing mostly retrospective series of FRI treatment in 3701 patients.

Treatment principles were described by five items. Diagnosis and classification, surgical protocol, treatment concept, follow-up protocol, and outcome, all showed a clear lack of consensus on diagnosis and treatment guidelines for FRI. This mirrors the situation for Prosthetic Joint Infection (PJI), identified many years ago [102].

It seems that basic management concepts of FRI treatment have not altered much since the beginning of the twentieth century [5], resulting in possible suboptimal care for FRI patients. The next section will discuss the discrepancies identified in the five specific domains.

### Diagnostic criteria and classification

This study shows that diagnostic criteria to define FRI were mentioned in 91% of all included studies, but no consensus exists on which parameters are relevant. Furthermore, only one study used a standardized definition for FRI (e.g., CDC guidelines) out of 47 studies using a definition. This is in line with conclusions of a previous review of 100 randomized clinical trials aimed at defining FRI [103].

A survey among 2327 orthopedic trauma surgeons in 2017 also confirmed that no consensus exists with respect to which diagnostic criteria should be used to define FRI and almost 90% of the respondents were convinced that a consensus-derived definition of FRI is urgently necessary [104].

Multiple time-related classifications were described in the literature that subdivide FRI into discrete groupings such as acute and chronic infections, or early, delayed and late onset infections [105–107].

These time windows are, to the best of our knowledge, not based on scientific evidence. This supports the view that they are poorly defined for FRI (e.g., time since injury, or time since onset of symptoms) and somewhat arbitrary (e.g., a 6 week transition from acute to chronic infection [108]). This review confirms that a variety of time windows is used to classify infection and that only 27 (29%) studies reported time in the classification of infection, suggesting that it is not a widely accepted parameter.

The aim of this review was to map all available diagnostic criteria and classifications used when describing patients treated for FRI. Given there are so many different views and no standardized criteria available, an equal comparison is not possible between studies. In the future this can and could be improved if researchers implement the recently developed consensus definition for FRI [108].

### Surgical protocols

This systematic review shows an enormous heterogeneity in treatment protocols. In 52 (56%) of all included studies, multiple anatomic locations are mentioned. Furthermore, different treatment strategies are describe (e.g., one-stage, two stage). This means that pooling of data related to outcome of these studies was not possible.

Twelve (13%) of the studies included, made use of a multidisciplinary approach to treat FRI. In these cases, a wide spectrum of team members were described, which also confirms the lack of consensus within this field. Recent literature shows that collaboration between multiple departments is essential to improve the outcome in FRI patients, not only with respect to treatment but also for prevention [109–114].

### Treatment specifications

As discussed before, this review identified many different treatment strategies for different types of FRI. As a consequence, different success rates were published. Due to heterogeneity it is almost impossible to compare outcome between studies. One explanation for this wide variety of published treatment strategies could be the lack of a consensus in the orthopedic trauma community regarding these strategies to manage FRI [103].

### Follow-up protocols

Protocolled follow-up of FRI patients was mentioned in 28% of all studies, showing a wide variety of parameters used in monitoring infection and functional outcome. No consensus exists on proper follow-up for this patient population, again emphasizing the need for international consensus. A recent overview confirms the importance of a well-defined follow-up in stratifications of patients [115].

### Surgical outcome

Included patients had a mean of three previous operations per patient before inclusion in the identified studies, further emphasizing the difficulty of FRI treatment. However, treatment failure and recurrence of disease rates found in this review occurred in 6–9% of all included cases, leading to amputation of the affected limb in 3–5%, which is a lower percentage than previously published [6–8]. This could be explained by underreporting of bad outcome, since surgeons tend to publish successful cases more easily than failures. Such publication bias is often seen in retrospective cohort series, which made up the majority of the series.

The patient-related outcome was calculated as a weighted mean of all included studies, including all available treatment options on different locations of FRI and different lengths of bone defects. Therefore, this outcome is in no way specific and it may only be seen as an overview of various patient-related outcomes in current studies.

### Limitations

This review was performed using a literature search with respect to FRI, based on terms that existed before a widely used definition of FRI was available. The lack of a definition makes it difficult to compare studies. In March 2018 a consensus definition of FRI from an international expert group has been published [108], finally offering the possibility to standardize reports and improve published literature.

In 66 (71%) of all studies no timescale was reported for infection, limiting the possibility to give a reliable overview of acute/early/delayed-onset FRI.

A better methodological quality in describing patient series is an absolute must, so comparison between series is possible ultimately leading to improvement of treatment.

We need to organize published work in such a way that comparison of either different surgical protocols or different types of FRI treated with the same surgical protocol is possible, further emphasizing the need for a uniform consensus on diagnosis and treatment of FRI.

Most series published do not provide information on individual patients, and do not supply median and standard deviation for any variable needed to perform statistical comparison between studies. To improve comparative analysis of the literature in the future, there is a need for a standard scientific notation of published data, ultimately leading to improvement in treatment strategies.

## Conclusion

This extensive literature review shows that the majority of studies report diagnostic criteria for FRI, and half of all studies describe FRI with a definition. There is an enormous heterogeneity of disease variation in and between reported patient series, surgical treatment protocols, and a lack of follow-up reports. This confirms the lack of standardized guidelines with respect to diagnosis and treatment of FRI, and mimics the situation for PJI identified many years ago. Standardized reporting and internationally accepted guidelines are urgently required to improve research and ultimately the quality of care for patients suffering from this significant and sometimes devastating complication.

## Appendix 1: Search terms used for the individual databases

- **Medline OVID**: (“osteitis”/ OR “osteomyelitis”/ OR (osteitis OR osteomyelitis).ab,ti.) AND (exp “Fractures, Bone”/ OR (fracture* OR nonunion* OR malunion* OR nonunited* OR malunited* OR posttraum* OR post-traum*).ab,ti.) AND (“Surgical Procedures, Operative”/ OR “debridement”/ OR osteitis/su OR osteomyelitis/su OR (surger* OR surgic* OR debridement*).ab,ti.) AND (“observational study”/ OR exp “Cohort Studies”/ OR “Case-Control Studies”/ OR “Cross-Sectional Studies”/ OR “multicenter study”/ OR “comparative study”/ OR “clinical study”/ OR exp “clinical trial”/ OR “Random Allocation”/ OR exp “treatment outcome”/ OR (((observation* OR comparativ*) ADJ6 (stud* OR data OR research)) OR cohort* OR longitudinal* OR retrospectiv* OR prospectiv* OR ((case OR cases OR match*) ADJ3 control*) OR (cross ADJ section*) OR correlation* OR multicenter* OR multi-center* OR follow-up* OR followup* OR clinical* OR trial OR random* OR (treatment ADJ3 (outcome* OR fail* OR success*))).ab,ti.) NOT (letter OR news OR comment OR editorial OR congresses OR abstracts).pt. AND english.la.
- **Embase**: (‘osteitis’/de OR ‘osteomyelitis’/de OR ‘chronic osteomyelitis’/de OR (osteitis OR osteomyelitis):ab,ti) AND (‘fracture’/exp OR ‘posttraumatic complication’/de OR (fracture* OR nonunion* OR malunion* OR nonunited* OR malunited* OR posttraum* OR post-traum*):ab,ti) AND (‘surgery’/de OR ‘surgical technique’/de OR ‘debridement’/de OR ‘osteitis’/exp/dm_su OR (surger* OR surgic* OR debridement*):ab,ti) AND (‘observational study’/exp OR ‘cohort analysis’/exp OR ‘longitudinal study’/exp OR ‘retrospective study’/exp OR ‘prospective study’/exp OR ‘case control study’/de OR ‘cross-sectional study’/de OR ‘correlational study’/de OR ‘major clinical study’/de OR ‘multicenter study’/de OR ‘comparative study’/de OR ‘follow up’/de OR ‘clinical study’/de OR ‘clinical article’/de OR ‘clinical trial’/exp OR ‘randomization’/exp OR ‘intervention study’/de OR ‘open study’/de OR ‘treatment outcome’/exp OR (((observation* OR comparativ*) NEAR/6 (stud* OR data OR research)) OR cohort* OR longitudinal* OR retrospectiv* OR prospectiv* OR ((case OR cases OR match*) NEAR/3 control*) OR (cross NEXT/1 section*) OR correlation* OR multicenter* OR multi-center* OR follow-up* OR followup* OR clinical* OR trial OR random* OR (treatment NEAR/3 (outcome* OR fail* OR success*))):ab,ti) NOT ([Conference Abstract]/lim OR [Letter]/lim OR [Note]/lim OR [Editorial]/lim) AND [english]/lim
- **Web of Science**: TS=(((osteitis OR osteomyelitis)) AND ((fracture* OR nonunion* OR malunion* OR nonunited* OR malunited* OR posttraum* OR post-traum*)) AND ((surger* OR surgic* OR debridement*)) AND ((((observation* OR comparativ*) NEAR/5 (stud* OR data OR research)) OR cohort* OR longitudinal* OR retrospectiv* OR prospectiv* OR ((case OR cases OR match*) NEAR/2 control*) OR (cross NEAR/1 section*) OR correlation* OR multicenter* OR multi-center* OR follow-up* OR followup* OR clinical* OR trial OR random* OR (treatment NEAR/2 (outcome* OR fail* OR success*))))) AND DT=(article) AND LA=(english)
- **Cochrane**: ((osteitis OR osteomyelitis):ab,ti) AND ((fracture* OR nonunion* OR malunion* OR nonunited* OR malunited* OR posttraum* OR post-traum*):ab,ti) AND ((surger* OR surgic* OR debridement*):ab,ti)
- **Google scholar**: “posttraumatic|traumatic osteitis|osteomyelitis” surgery|surgical|debridement allintitle:”posttraumatic|traumatic osteitis|osteomyelitis” surgery|surgical|debridement
